# Study of Interference Detection of Rail Transit Wireless Communication System Based on Fourth-Order Cyclic Cumulant

**DOI:** 10.3390/s23198291

**Published:** 2023-10-07

**Authors:** Peng Wang, Junliang Yao, Yong Pu, Shuyuan Zhang, Lu Wen

**Affiliations:** 1 China Railway First Survey and Design Institute Group Co., Ltd., Xi’an 710043, China; th_wangpeng6.yy@crcc.cn; 2 School of Automation and Information Engineering, Xi’an University of Technology, Xi’an 710048, China; 17726694754@163.com (Y.P.); zsyxaut@163.com (S.Z.); 3 State Key Laboratory of Rail Transit Engineering Informatization (FSDI), Xi’an 710043, China; wenlu.yy@crcc.cn

**Keywords:** rail transit wireless communications, interference detection, cyclic cumulant, multipath propagation, Doppler effect

## Abstract

The wireless communication system is used to provide dispatching, control, communication and other services for rail transit operations. In practice, interference from other wireless communication systems will affect the normal operation of trains, so it is an urgent problem to study the interference detection algorithms for rail transit applications. In this paper, the fourth-order cyclic cumulant (FOCC) of signals with different modulation modes is analyzed for the narrow-band wireless communications system of rail transit. Based on the analysis results, an adjacent-frequency interference detection algorithm is proposed according to the FOCC of the received signal within the predetermined cyclic frequency range. To detect interference with the same carrier frequency, a same-frequency interference detection algorithm using the relationship between the FOCC and the received power is proposed. The performance of the proposed detection algorithms in terms of correct rate and computational complexity is analyzed and compared with the traditional second-order statistical methods. Simulation results show that when an interference signal coexists with the expected signal, the correct rates of the adjacent-frequency and the same-frequency interference detection algorithms are greater than 90% when the signal-to-noise ratio (SNR) is higher than 2 dB and −4 dB, respectively. Under the practical rail transit wireless channel with multipath propagation and the Doppler effect, the correct rates of the adjacent-frequency and the same-frequency interference detection algorithms are greater than 90% when the SNR is higher than 3 dB and 7 dB, respectively. Compared with the existing second-order statistical methods, the proposed method can detect both the adjacent-frequency and the same-frequency interference when the interference signals coexist with the expected signal. Although the computational complexity of the proposed method is increased, it is acceptable in the application of rail transit wireless communication interference detection.

## 1. Introduction

Urban rail transit is a high-capacity and high-efficiency mode of public transportation. In recent years, China’s rail transit industry has achieved rapid development [1]. By the end of 2021, a total of 9192.62 km of urban rail transportation routes were in operation in more than 50 cities, 78.9% of which are metro lines. As an important component of the urban rail transit system, wireless communication not only connects various types of equipment but also provides effective control to ensure the normal operation of the train. Currently, the main standards and wireless frequency spectrum used in urban rail transit mainly include 800 MHz TETRA (Terrestrial Trunked Radio) [2] used for vehicle-to-ground wireless communication trunk dispatching systems; 2.4 GHz and 5.8 GHz WiFi used for passenger information systems [3]; and 1.8 GHz LTE-M (LTE-Machine-to-Machine) [4] used for communication-based train control (CBTC) systems [5]. Among them, 2.4 GHz and 5.8 GHz are public frequency bands, which are inevitably interfered with by external user’s equipment. The 1.8 GHz LTE-M is a specially used system. In addition to rail transit, it is also used in industries such as electric power, airports, petroleum, etc. Therefore, it could be interfered with by the same-frequency signal. What is more, the 1.8 GHz signal will also be interfered with by the telecom operators’ adjacent-frequency systems, such as the China Telecom FDD-LTE and China Mobile DCS1800 systems. If the interference power exceeds a certain threshold, the communication service will be interrupted, and the train will be emergency braked. Therefore, interference signal detection is important to ensure the safe and stable operation of trains [6,7].

Classical interference detection algorithms include time-domain energy detection [8,9,10], frequency-domain detection [11,12,13], time–frequency-domain detection [14,15], array-based signal processing [16], etc. Guo proposed a blind interference detection algorithm based on an energy detector [10], which is very simple and widely used. However, the performance of the algorithm is greatly affected by the estimation of the noise power, and the estimation error will cause the algorithm’s accuracy to decrease. In addition, the time-domain method lacks information on the interference frequency and cannot locate the interference source, so it is not effective to eliminate the interference of the rail transit wireless communication system. The continuous mean elimination (CME) algorithm for interference detection based on frequency-domain analysis has been proposed [12], which sets the detection threshold based on minimizing the false-alarm probability. The frequency-domain method can not only judge the presence of interference signals but also obtain the location information of interference frequency points, which makes up for the deficiency of the time-domain method. However, the CME algorithm requires a large carrier frequency interval between the interference signal and the expected signal, so it is unable to detect the same-frequency interference that often occurs in the rail transit wireless communication system.

In fact, it is not enough to analyze the characteristics of the time domain or frequency domain independently, and the relationship between them should also be explored. With the help of Wigner–Ville distribution [17] and short-time Fourier transform, a time–frequency-domain-based interference detection method is proposed [18], but the algorithm complexity is too high for online use. In addition, array signal processing methods can also be used for interference detection in multi-antenna systems [19,20]. Due to the limited number of antenna elements in railway communication terminals, the traditional array signal processing method cannot be applied.

To address these problems, the single-channel source number estimation and signal separation algorithms based on a higher-order cyclic cumulant are presented in [21,22]. With only one antenna at the receiver, the observation matrix is represented by a single parameter, and an evaluation function is designed using the properties of the high-order cumulant to obtain the optimal value of this parameter [21]. After recognizing the observation matrix, four effective feature parameters of the high-order cumulant were extracted, and single-channel time-frequency overlapping signals were separated through signal reconstruction [22]. The method has good anti-noise performance and does not require estimation of the prior information such as noise power, so it is suitable for interference detection in rail transit wireless communications. In this paper, a fourth-order cyclic cumulant (FOCC)-based interference detection method is proposed. First, the single-channel time-frequency overlapping signal model for rail transit downlink communication is described. Second, the detection algorithms for both the adjacent-frequency and the same-frequency interference are implemented. By calculating the FOCC of the received signal and judging the amplitude of the cyclic cumulant at different cyclic frequencies, the interference signals can be detected. Finally, simulation tests under typical rail transit wireless channels, such as two paths and large Doppler frequencies, are performed to verify the effectiveness of the proposed methods. We highlight our main contributions in the following summary of the paper:

- Two FOCC-based interference detection algorithms are proposed for application in the train transit wireless communication scene, which is susceptible to both adjacent-frequency interference and same-frequency interference.
- The correct rate and computational complexity of the proposed algorithms are analyzed and compared with the traditional second-order statistical methods.
- The impact of multipath and Doppler on algorithm performance is analyzed through simulation tests.
- The advantages of the proposed algorithms include the following:
  (1) The FOCC of the received time-frequency overlapping signal is used as a feature for interference detection. Compared with the traditional second-order statistical properties, the anti-noise performance is better because it is 0 for the cyclic cumulant of white Gaussian noise to be larger than the second order. This is very useful in rail transit wireless communication applications because the background noise varies with the arrival and departure of trains.
  (2) Traditional time-domain energy detectors are only effective during quiet periods, i.e., they can only detect interference when expected signals are not present, and the detection performance largely depends on the accuracy of the noise estimation. However, in trail transit wireless communication scenarios, the interference signals usually coexist with the expected signal, and there are errors in estimating the noise variance. In this case, the energy detectors would no longer be applicable, and the proposed method in this paper has significant advantages over the existing one.
  (3) The traditional frequency-domain method can only detect adjacent-frequency interference. The proposed methods can detect both adjacent-frequency interference and same-frequency interference because the spectral power of the expected signal is considered to detect same-frequency interference.
  (4) In a rail transit wireless communication scenario, the multipath propagation and Doppler effect will lead to the performance degradation of interference detection algorithms. The proposed FOCC-based method can overcome the Doppler effect because its influence on the cyclic frequencies can be ignored. Although the proposed method is also affected by multipath propagation, it still works well at medium SNRs.

## 2. Signal Model

In this section, we define the signal received by the downlink communication as a single-channel time-frequency overlapping signal, since the terminal is usually equipped with a single antenna in the rail transit wireless communication. In practice, rail traffic has a dedicated frequency band for communication, so the probability of multiple interference sources occurring simultaneously is low. In this paper, it is assumed that the maximum number of source signals will not be more than three; then, the received signal can be expressed as follows:

(1) $$x(t)=\sum_{i=1}^{r}s_{i}(t)+n(t)$$

where *r* ≤ 3 is the number of source signals, and $s_{i}(t)=A_{i}(t)e^{j\left(2\pi f_{c}{}_{i}t+\theta_{i}(t)+\phi_{i}\right)}$ represents the *i*th source signal, where $A_{i}(t)$, $f_{ci}$, $\theta_{i}(t)$ and $\phi_{i}$ are the signal amplitude, carrier frequency, modulation phase and initial phase, respectively. *n*(*t*) is the additive white Gaussian noise.

Due to the complexity of the railway wireless communications environment, the influence of multipath and Doppler on interference detection should also be considered. In the presence of the multipath, the received signal contains multiple signals at the same frequency and at different power levels, making it more difficult to detect interfering signals.

Multipath signals have different carrier phases and delays corresponding to the main path signal, and the received signal through a multipath fading channel can be modeled as follows [23]:

(2) $$x(t)=\sum_{i=1}^{r}\sum_{l=1}^{L}h_{l}s_{i}(t-\tau_{l})e^{j2\pi f_{d}t}+n(t)$$

where *L* is the number of paths, and $h_{l}$ and $\tau_{l}$ are the attenuation coefficient and the time delay corresponding to path *l* from the transmitter to the receiver. $f_{d}=f_{c}\cdot v/c$ is the Doppler shift, where *v* is the speed at which the train is traveling with an average speed of 30 m/s, and *c* is the speed of light with a magnitude of $3\times 10^{8}\;m/s$. For simplicity, this paper assumes that the carrier synchronization of the received signal *x*(*t*) is perfect, and only the strongest path information is considered, ignoring other weaker path signals; that is, the following research considers only the two-path situation for the detection of interfering signals.

## 3. Algorithm Principle and Implementation

### 3.1. Fourth-Order Cyclic Cumulant

In this section, the FOCC of various modulated signals will be derived. According to the definition, the *k*th-order cyclic moment of the cyclic smooth signal x(i),i=0,1,…,N−1 is calculated as follows [24]:

(3) $$\begin{array}{l} M_{kx}^{\alpha }(\tau_{1},\tau_{2},\ldots ,\tau_{k-1})\\ =\frac{1}{T}\sum_{i=0}^{N-1}x(i)x(i+\tau_{1})\cdots x(i+\tau_{k-1})e^{-j2k\pi \alpha t}\\ ={\langle x(i)x(i+\tau_{1})\cdots x(i+\tau_{k-1})e^{-j2k\pi \alpha t}\rangle }_{t} \end{array}$$

where *k* is the order of the cyclic moment, $\tau_{1},\tau_{2},\ldots ,\tau_{k-1}$ is the fixed time delay, and *α* is the cyclic frequency. *N* is the number of sample points during the time period *T*, and ${\langle \cdot \rangle }_{t}$ represents the time averaging operation. According to the transfer relation between the cyclic moment and the cyclic cumulant, the FOCC $C_{40,x}^{\alpha }(\tau_{1},\tau_{2},\tau_{3})$ of the signal *x*(*t*) can be expressed as [24]

(4) $$\begin{array}{cc} C_{40,x}^{\alpha }(\tau_{1},\tau_{2},\tau_{3}) & =M_{4x}^{\alpha }(\tau_{1},\tau_{2},\tau_{3})\\ & -M_{2x}^{\alpha }(\tau_{1})M_{2x}^{\alpha }(\tau_{3}-\tau_{2})\\ & -M_{2x}^{\alpha }(\tau_{2})M_{2x}^{\alpha }(\tau_{1}-\tau_{3})\\ & -M_{2x}^{\alpha }(\tau_{3})M_{2x}^{\alpha }(\tau_{2}-\tau_{1}) \end{array}$$

when $\tau_{1}=\tau_{2}=\tau_{3}=0$,

(5) $$\begin{array}{cc} C_{40,x}^{\alpha }(0,0,0) & =M_{4x}^{\alpha }(0,0,0)-3M_{2x}^{\alpha }(0)M_{2x}^{\alpha }(0)\\ & ={\langle x^{4}(t)e^{-j8\pi \alpha t}\rangle }_{t}-3{\langle x^{2}(t)e^{-j4\pi \alpha t}\rangle }_{t}{}^{2} \end{array}$$

Since it is 0 for the cyclic cumulant of white Gaussian noise to be larger than the second order, the FOCC of the noise in Equation (1) is 0. Therefore, the method has better anti-noise performance. Then, the FOCC of the time-frequency overlapping signal in Equation (1) is

(6) $$\begin{array}{cc} C_{40,x}^{\alpha }(0,0,0) & =\sum_{i=1}^{r}C_{40,s_{i}}^{\alpha }(0,0,0)+C_{40,n}^{\alpha }(0,0,0)=\sum_{i=1}^{r}C_{40,s_{i}}^{\alpha }(0,0,0)\\ & =\sum_{i=1}^{r}{\langle s_{i}{}^{4}(t)e^{-j8\pi \alpha t}\rangle }_{t}-\sum_{i=1}^{r}3{\langle s_{i}{}^{2}(t)e^{-j4\pi \alpha t}\rangle }_{t}{}^{2}\\ & =\sum_{i=1}^{r}{\langle A_{i}{}^{4}(t)e^{j4(\pi (2f_{ci}-2\alpha )t+\theta_{i}+\phi_{i})}\rangle }_{t}-\sum_{i=1}^{r}3{\langle A_{i}{}^{2}(t)e^{j2(\pi (2f_{ci}-2\alpha )t+\theta_{i}+\phi_{i})}\rangle }_{t}{}^{2} \end{array}$$

When the cyclic frequency α is equal to the carrier frequency $f_{ci}$ of the *i*th signal component, the signal modulation is BPSK, QPSK, 8 QAM or 16 QAM, and the initial phase $\phi_{i}$ obeys the uniform distribution in the range of [0, 2π], Equation (6) can be derived as

(7) $$\begin{array}{cc} C_{40,x}^{\alpha =f_{ci}}(0,0,0) & ={\langle A^{4}(t)e^{j4(\pi (2f_{ci}-2\alpha )t+\theta (t)+\phi )}\rangle }_{t}-3{\langle A^{2}(t)e^{j2(\pi (2f_{ci}-2\alpha )t+\theta (t)+\phi )}\rangle }_{t}^{2}\\ & =A^{4}e^{j4\phi }{\langle e^{j4\theta (t)}\rangle }_{t}-3A^{2}e^{j4\phi }{\langle e^{j2\theta (t)}\rangle }_{t}^{2}\\ & =\left\{\begin{array}{c} -2A^{4}e^{j4\phi },\;\mathrm{BPSK}\\ \;A^{4}e^{j4\phi },\;\mathrm{QPSK}\\ 0.34A^{4}e^{j4\phi },\;8\mathrm{QAM}\\ 0.68A^{4}e^{j4\phi },\;16\mathrm{QAM} \end{array}\right. \end{array}$$

For the cyclic frequency not equal to the carrier frequency, Equation (6) can be derived as

(8) $$\begin{array}{cc} C_{40,x}^{\alpha \neq f_{c}{}_{i}}(0,0,0) & ={\langle A_{i}^{4}(t)e^{j4(\pi (2f_{c}{}_{i}-2\alpha )t+\theta_{i}(t)+\phi_{i})}\rangle }_{t}-3{\langle A_{i}^{2}(t)e^{j2(\pi (2f_{c}{}_{i}-2\alpha )t+\theta_{i}(t)+\phi_{i})}\rangle }_{t}^{2}\\ & ={\langle A_{i}^{4}(t)e^{j4\phi_{i}}\cdot e^{j8\pi (f_{c}{}_{i}-\alpha )t+4\theta_{i}(t)}\rangle }_{t}-3{\langle A_{i}^{2}(t)e^{j2\phi_{i}}\cdot e^{j4\pi (f_{c}{}_{i}-\alpha )t+2\theta_{i}(t)}\rangle }_{t}^{2}\\ & =0 \end{array}$$

**Figure 1:**
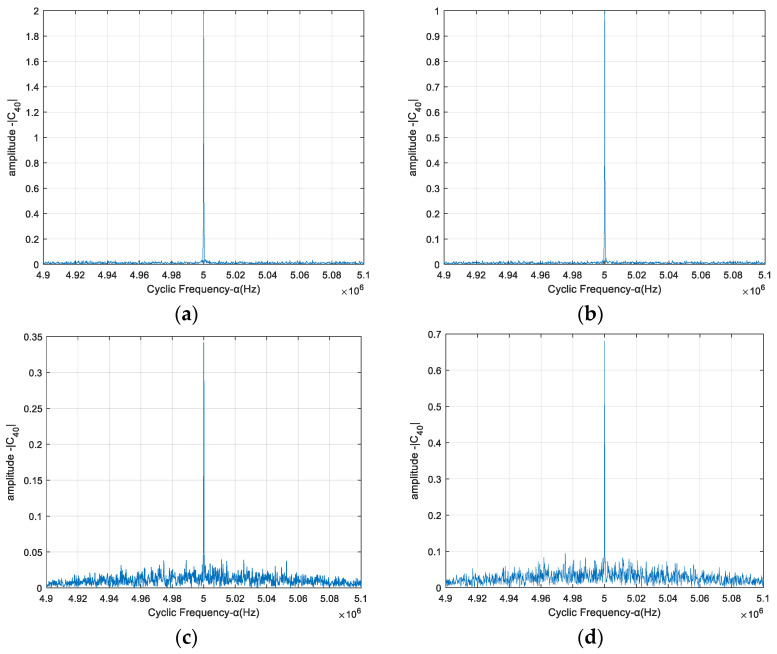
Amplitude spectrum of the FOCC of a single modulated signal. (**a**) BPSK, (**b**) QPSK, (**c**) 8 QAM, (**d**) 16 QAM.

Figure 1a–d show the amplitude spectrum $\left|C_{40,x}^{\alpha }(0,0,0)\right|$ with respect to the cyclic frequency *α* for BPSK, QPSK, 8 QAM and 16 QAM modulated signals, respectively. The carrier frequencies of the modulated signals are 5 MHz, the symbol rate is 0.5 Mbps, and the SNR is 4 dB.

We can see from Figure 1 that the amplitude is not zero when the cyclic frequency is equal to the carrier frequency, and the amplitude values are different for different modulation modes. The amplitudes of the FOCC of a single BPSK, QPSK, 8 QAM and 16 QAM modulated signal are 2, 1, 0.34 and 0.68, respectively. When the cyclic frequency is not equal to the carrier frequency, the amplitude of FOCC is close to zero. These results are consistent with the theoretical analysis of Equation (7), which provides a basis for judging the number of mixed signals.

### 3.2. Detection Algorithm for the Adjacent-Frequency Interference 

To detect the adjacent-frequency interference, the source signals with different carrier frequencies are mixed, and the FOCC of the mixed signal is analyzed.

**Figure 2:**
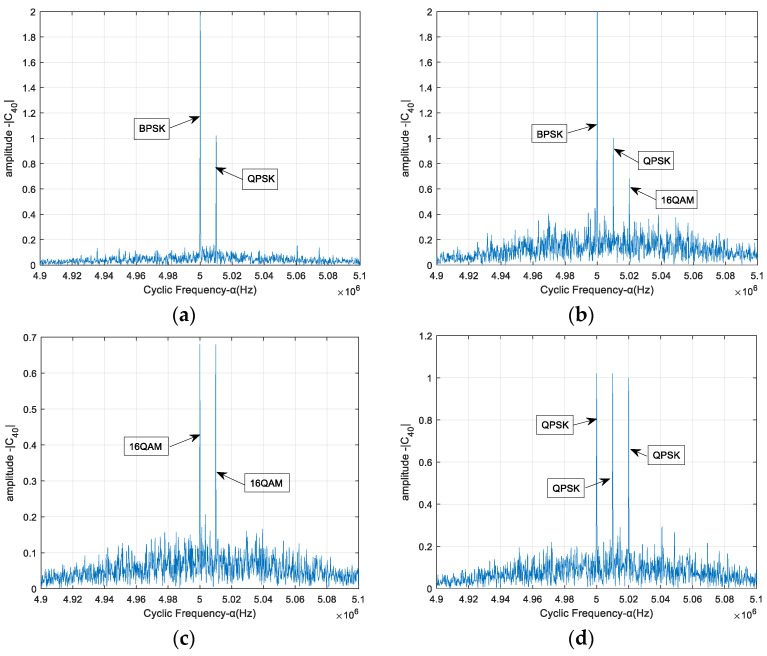
Amplitude spectrum of FOCC of mixed signals with different carrier frequencies. (**a**) BPSK&QPSK, (**b**) BPSK&QPSK&16 QAM, (**c**) 16 QAM&16 QAM, (**d**) QPSK&QPSK&QPSK.

In the case of *r* = 2, Figure 2a shows the FOCC amplitude of the time-frequency overlapping signals with respect to the cyclic frequency. The modulation modes of two source signals are BPSK and QPSK, and the carrier frequencies are 5 MHz and 5.01 MHz, respectively. The symbol rates of both source signals are 0.5 Mbaud, and the SNR is 4 dB. We can see that, when the carrier frequencies are different, the FOCCs of the mixed signals have discrete spectral lines at the carrier frequencies of each source. The amplitudes for the BPSK and QPSK signals are similar to the results in Figure 1. The amplitudes at non-carrier frequencies are slightly higher than the results in Figure 1 due to the mutual interference between two source signals. In the case of *r* = 3, the modulation modes of three source signals are BPSK, QPSK and 16 QAM, and the carrier frequencies are 5 MHz, 5.01 MHz and 5.02 MHz, respectively. From Figure 2b, we can see that the amplitudes at carrier frequencies are similar to the results in Figure 2a. The amplitudes at non-carrier frequencies are higher than those in Figure 2a because the mutual interference increases. The FOCCs of two sources with the same modulation order 16 QAM and different carrier frequencies are shown in Figure 2c, and the FOCCs of three sources with the same modulation order QPSK and different carrier frequencies are shown in Figure 2d. We can see that, when the carrier frequencies are different, the FOCCs of the mixed signals have discrete spectral lines at the carrier frequencies of each source. The amplitudes for each signal are similar to the results in Figure 1.

Based on the above analysis results, the adjacent-interference detection algorithm is proposed by determining the number of discrete spectral lines in the amplitude spectrum. The adjacent-frequency interference exists when the number of discrete spectral lines is not equal to the number of the expected signal, which is known in advance. The steps of the algorithm are described as follows (Algorithm 1).
**Algorithm 1** FOCC-based Adjacent-Frequency Interference Detection Algorithm(Step 1) The power of the received mixed signal *x*(*t*) is normalized, and the power spectrum is calculated to determine the search range of the cyclic frequency α. (Step 2) The FOCC of *x*(*t*) is calculated, and its amplitude spectrum for cyclic frequencies $\alpha -|C_{40,x}^{\alpha }(0,0,0)|$ is obtained. (Step 3) A suitable threshold is set to calculate the number of discrete spectral lines in the amplitude spectrum. In this paper, the threshold is set to 0.5 according to the analysis of Equation (7). (Step 4) The adjacent-frequency interference occurs when the number of discrete spectral lines is greater than the number of expected signals; otherwise, there is no adjacent-frequency interference.

### 3.3. Detection Algorithm for the Same-Frequency Interference

For adjacent-frequency interference, the FOCCs of different carrier frequencies are separated. The above-proposed algorithm is not applicable to the detection of same-frequency interference because the carrier frequencies of the expected and the interference signals are the same and there is only one discrete spectral line in the amplitude spectrum. In this subsection, the relationship between the FOCC and the received power is analyzed, which is used to design the detection algorithm for the same-frequency interference.

In practice, the power of the received signal can be obtained, and the case of one interference signal is considered in the analysis. The case of more interference signals is similar. We denote the received power as *k = k*_1_ *+ k*_2_, where *k*_1_ is the power of the expected signal, and *k*_2_ is the power of the interference signal. Assuming that the modulation of the expected signal is known in advance, the FOCCs $C_{40,x}^{\alpha }\left(0,0,0\right)$ of the received signal *x*(*t*) at the cyclic frequency $\alpha =f_{ci}$ are shown in Table 1.

We can see that if the received power is fixed as *k*, $C_{40,x}^{\alpha =f_{ci}}$ varies with the interference power *k*_2_ and the modulation modes. If the expected signal is BPSK modulation, and the FOCC without interference is larger than the FOCC with interference, then an appropriate threshold can be set to distinguish whether there is interference. If $C_{40,x}^{\alpha =f_{ci}}$ is greater than the threshold, there is no interference, and vice versa. Considering the noise effect, the threshold $\sigma_{1}$ can be designed according to the noise power, and the interference detection is given by

(9) $$\mathrm{Expected}\;\mathrm{signal}\;\mathrm{is}\;BPSK\Rightarrow \left\{\begin{array}{l} H_{0},\left(C_{40,x}^{\alpha =f_{ci}}>2k^{2}-\sigma_{1}\right)\\ H_{1},\left(otherwise\right) \end{array}\right.$$

In Equation (9), *H*_1_ represents the presence of interference, and *H*_0_ represents no interference. Considering the noise influence and the decision distance in the first row of Table 1, the threshold $\sigma_{1}$ in Equation (9) is set to 0.5 in the following algorithm simulation.

If the expected signal is QPSK modulation, the FOCC without interference $C_{40,x}^{\alpha =f_{ci}}=k^{2}$ is not always the largest. Similarly, a threshold $\sigma_{2}$ can be designed, and the interference detection is given by

(10) $$\mathrm{Expected}\;\mathrm{signal}\;\mathrm{is}\;QPSK\Rightarrow \left\{\begin{array}{l} H_{0},\left(k^{2}-\sigma_{2}<C_{40,x}^{\alpha =f_{ci}}<k^{2}+\sigma_{2}\right)\\ H_{1},\left(otherwise\right) \end{array}\right.$$

The meanings of *H*_0_ and *H*_1_ are the same as those described after Equation (9). The threshold $\sigma_{2}$ in Equation (10) is set to 0.1 in the following algorithm simulation.

The procedure for deriving the FOCCs for BPSK/QPSK/16 QAM modulated signals is given above. For received signals with multipath Doppler effects, the signal model is shown in Equation (2), and the FOCC of the received signal with multipath Doppler effects is given by

(11) $$\begin{array}{cc} C_{40,x}^{\alpha }(0,0,0) & ={\langle {\left(\sum_{i=1}^{2}\sum_{l=1}^{2}h_{i}s_{i}(t)e^{j2\pi f_{di}t}\right)}^{4}e^{-j8\pi \alpha t}\rangle }_{t}-3{\langle {\left(\sum_{i=1}^{2}\sum_{l=1}^{2}h_{i}s_{i}(t)e^{j2\pi f_{di}t}\right)}^{2}e^{-j4\pi \alpha t}\rangle }_{t}^{2}\\ & ={\langle \sum_{i=1}^{2}\sum_{l=1}^{2}h_{i}{}^{4}A^{4}a^{4}(t)e^{j8\pi (f_{ci}+f_{di}-\alpha )t}\rangle }_{t}-3{\langle \sum_{i=1}^{2}\sum_{l=1}^{2}h_{i}{}^{2}A^{2}a^{2}(t)e^{j4\pi (f_{ci}+f_{di}-\alpha )t}\rangle }_{t}^{2} \end{array}$$

From Equation (11), it can be seen that when the cyclic frequency is equal to the sum of the carrier frequency and the Doppler frequency, there is a non-zero value of the FOCC of the received signal. Since the Doppler frequency is much smaller than the carrier frequency, i.e., $f_{c}+f_{d}\approx f_{c}$, we see that the FOCC value is not zero when the cyclic frequency is equal to the carrier frequency. However, due to the multipath, the amplitude of FOCC changes, and simultaneously, the signal power decreases, but the overall nature of the FOCC does not change. In summary, the presence of a multipath Doppler effect in the received signal does not significantly alter the nature of the FOCC, making it feasible to use the FOCC for interference detection, and subsequent simulations have confirmed this conclusion.

### 3.4. Computational Complexity

In this subsection, the computational complexity of the proposed method is analyzed and compared with the traditional interference detection algorithms based on second-order statistical properties. The time complexity, which describes the number of elementary operations needed to perform a method, is used to measure the computational complexity. In general, we focus on the asymptotic behavior of the computational workload as the input size increases. The time complexity *T*(*N*) is usually expressed as *T*(*N*) = *O*(*N*), where *N* is the input size. To be impartial, the time complexity of the three methods is analyzed below.

The computational complexity of all algorithms includes statistics calculation complexity and interference decision complexity. The computational complexity comparison of the three methods is shown in Table 2. For the proposed FOCC-based algorithm, the statistics calculation process is shown in Equation (5), and the input sizes are the number of symbols in one data frame and the length of the cyclic frequency. If *N* is the number of symbols, and *M* is the length of the cyclic frequency, then the statistics calculation complexity is *O*(*MN*^5^). To detect an interference, a suitable threshold is preset, and the interference decision complexity is *O*(*M*). For traditional second-order statistical methods, the energy detector in Ref. [10] and the frequency-domain CME algorithm in Ref. [12] are considered. In the energy detector, the energy of the received signal is calculated; then, the statistics calculation complexity is *O*(*N*^2^). To detect an interference, the noise variance is estimated to set the threshold, and the interference decision complexity is *O*(*N*^2^). In the frequency-domain CME algorithm, the fast Fourier transform (FFT) of the received signal is performed, and the statistics calculation complexity is *O*(*N*log*N*). To detect an interference, the continuous mean elimination with *P* iterations is performed, and the interference decision complexity is *O*(*PN*).

In practice, the variables mentioned above usually satisfy 500≤N≤1000, 100≤M≤200, and 100≤P≤500. From Table 2, we summarize that the proposed FOCC-based algorithm has higher statistics calculation complexity and lower interference decision complexity than the traditional methods. Although the total computational complexity is increased, it is acceptable in the application of rail transit wireless communication interference detection.

## 4. Simulation Experiments and Result Analysis

In this section, the proposed interference detection algorithms are evaluated using three simulation experiments, where the performance metric is the correct rate of the detection algorithms. In each experiment, 1000 independent trials are performed, and the correct rate is defined as the ratio of the number of correct trials to the total number of trials. 

In Experiment A, the correct rate of the proposed adjacent-frequency interference detection algorithm is simulated for different numbers of interference signals. The traditional energy detector method in Ref. [10] and the frequency-domain CME algorithm in Ref. [12] are also tested for comparison. In Experiment B, the correct rate of the proposed same-frequency interference detection algorithm is tested. In the rail transit scenario, the wireless signals will experience multipath propagation and the Doppler effect, due to reflections in station buildings and high-speed train movement. Therefore, the algorithm performances in a wireless channel with multipath and Doppler are evaluated in Experiment C. All the simulation experiments are implemented on a PC using MATLAB R2016a.

Experiment A. Correct rate of the adjacent-frequency interference detection algorithm

In this experiment, the algorithm described in Section 3.2 is simulated, and three source signals, with carrier frequencies 5 MHz/5.01 MHz/5.02 MHz and modulation modes BPSK/QPSK/16 QAM, are considered. To evaluate the performance of different numbers of interference signals, the received signal is mixed with any two or three source signals, where one is the expected source and the others are interference sources. In our simulation, there are 800 bits in one data frame, and the symbol rate is set to 0.5 Mbps for all the source signals. Assuming that the signal power is P_s_ = 1, and the SNR range is [−2:0.5:1, 2:1:8], the simulation results are obtained by averaging over 500 independent data frames. To illustrate the effect of different combinations of signals, three sets of signals with the same modulation and two sets of signals with different modulations were selected for testing.

The results in Figure 2 show that the FOCC values at the carrier frequencies are determined by the modulation modes of the signals. On the other hand, the FOCC values at the non-carrier frequencies depend on the noise and bit sequences. In order to estimate the number of carriers more accurately, the FOCC at the non-carrier frequencies is reduced by averaging over multiple calculations, assuming that the noise and bit sequences for each source have a random equal probability distribution. 

**Figure 3:**
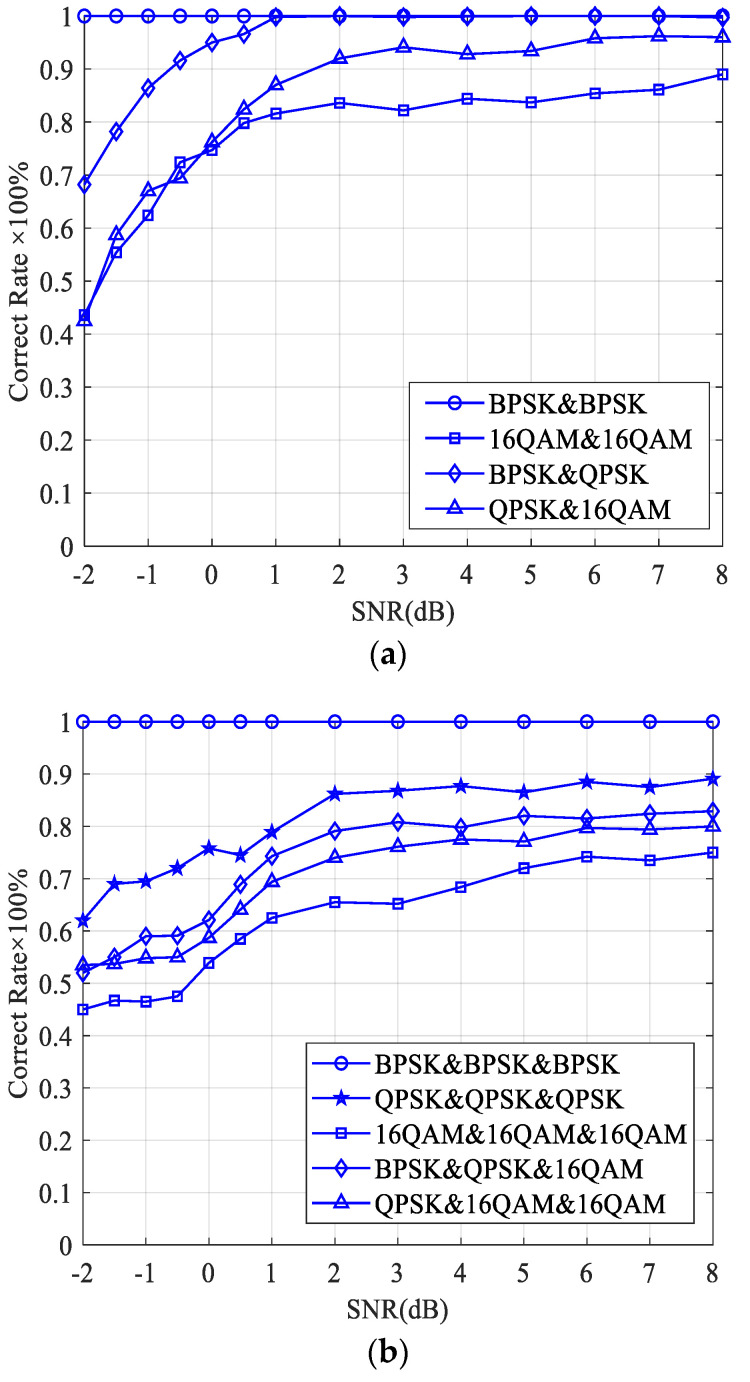
Correct rate of the adjacent-frequency interference detection algorithm. (**a**) One expected signal and one interference signal. (**b**) One expected signal and two interference signals.

The case of two sources, one being the expected signal and the other being the interference signal, is considered. The correct rate of the proposed adjacent-frequency interference detection algorithm is shown in Figure 3a. It is seen that the correct rate of the proposed method is greater than 90% when the SNR is higher than 2 dB, and the expected signal is BPSK or QPSK. The correct rate decreases at the same SNR when both the expected signal and the interference signal are 16 QAM because the value of the FOCC is affected by the modulation order. It is easier to see that the lower the modulation order, the higher the value of the FOCC. When the case of three sources, one being the expected signal and the other two being the interference signals, is considered, the correct rate of the proposed adjacent-frequency interference detection algorithm is shown in Figure 3b. It can be seen that as the number of overlapping signals increases, the correct rate of the proposed method decreases due to the greater influence of the interference term. The correct rate is 100% for BPSK mixed signals, and the results for QPSK and 16 QAM are 90% and 75%, respectively.

It is worth noting that the curves in Figure 3 are obtained by averaging over 500 independent trials. In each trial, the expected signal and interference signal are randomly selected from the source signals. Therefore, it may not be easy to distinguish the expected source and interference source in the legend of Figure 3. 

**Figure 4:**
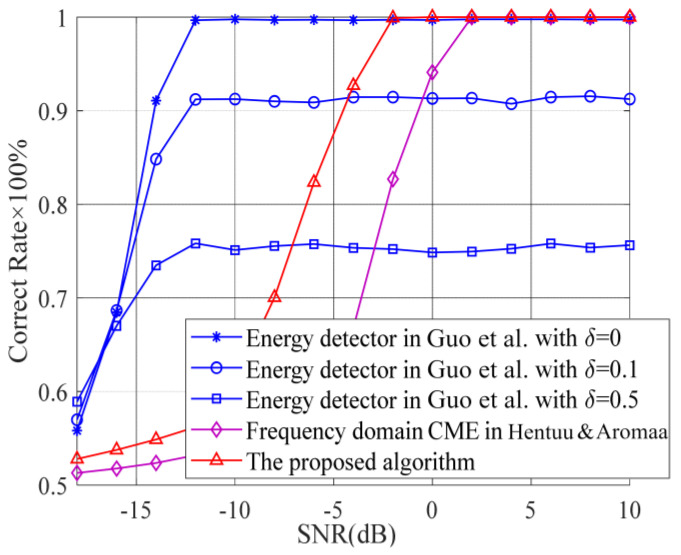
Correct rate comparison with only the interference signal. Guo et al., 2015 [10]; Hentuu & Aromaa, 2002 [12].

Because the energy detector is only suitable for interference detection during the quiet period when the expected signal is not present, in order to fairly compare the proposed algorithm with existing algorithms, we consider the situation that the interference signal occurs with a probability of 0.5, and the expected signal does not exist. The simulation results of the proposed adjacent-frequency interference detection algorithm, the time-domain energy detector in Ref. [10], and the frequency-domain CME algorithm in Ref. [12] are shown in Figure 4 for comparison.

The decision threshold of the energy detector is set based on the noise variance estimation, and the estimation error will deteriorate the detection performance. Assuming that the error of the noise variance is denoted by δ, the correct rate curves of the energy detector with δ=0, δ=0.1 and δ=0.5 are given in Figure 4 by the blue solid lines. We can see that the energy detector has a very good detection performance when δ=0. As the noise estimation error increases, the correct rate decreases significantly due to the deviation of the decision threshold. For both the frequency-domain CME algorithm and the proposed algorithm, the correct rates are greater than 50% even in the low-SNR region because they can always correct when there is no interference. The correct rate of the proposed algorithm reaches 100% when SNR = −2 dB, which is 4 dB lower than the result of the frequency-domain CME algorithm.

As we can see from Figure 4, the proposed method has a poor performance compared to a traditional energy detector in the low-signal-to-noise ratio region. However, the energy detector is only suitable for detecting the interference signal during the quiet period, and the algorithm performance is deteriorated by the noise variance estimation error. The proposed method has a wider applicability and does not require estimation of noise variance.

Experiment B. Correct rate of the same-frequency interference detection algorithm

**Figure 5:**
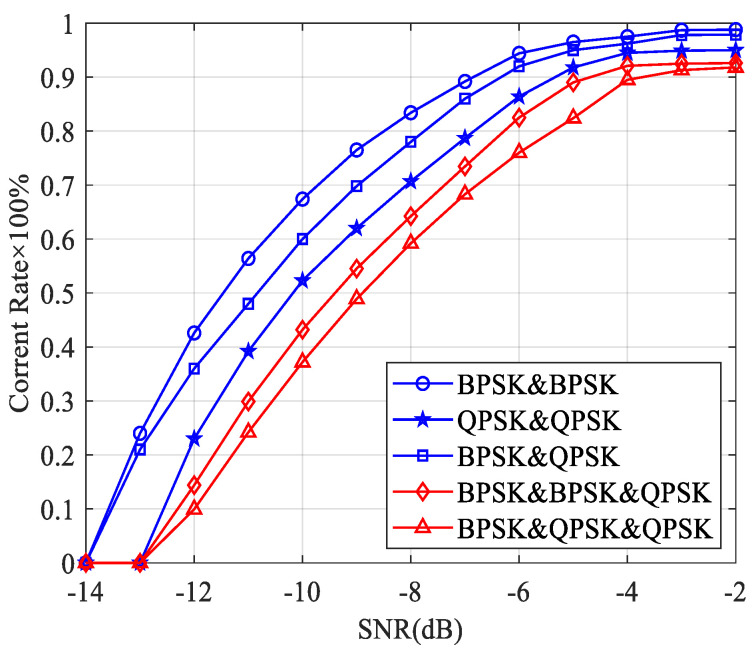
Correct rate of the same-frequency interference detection algorithm.

In this experiment, the algorithm described in Section 3.3 is simulated. Three source signals, with the same carrier frequency of 5 MHz are considered. The received signal contains one expected signal and no more than two interference signals. The power of each signal is random, and the sum power of the received signal is normalized to 1. The other experimental conditions are identical to Experiment A, and the results are shown in Figure 5.

We can see from Figure 5 that the correct rate of the same-frequency interference detection is greater than 90% when the SNR is higher than −4 dB, which is better than the results of the adjacent-frequency interference detection in Figure 3. This is because the adjacent-frequency interference signals have a different frequency component compared with the expected signal, resulting in an interference term between signals. The interference term will cause the FOCC to have a non-zero value at the non-cyclic frequency, which will affect the spectral line detection at the cyclic frequencies. In the case of same-frequency interference, the FOCC of the received signal at the cyclic frequency is not affected by other frequency components, and the relationship between the received signal power and the FOCC is used to limit the influence of the interference term.

Experiment C. Interference detection in multipath and Doppler environments

In the rail transit scenario, such as a subway or high-speed train, the wireless channel is mostly single-path or two-path. Meanwhile, the received signal suffers from the Doppler effect when the train is running at high speed [25]. Therefore, it is useful to analyze the performance of interference detection algorithms in multipath and Doppler environments. In this experiment, a two-path propagation channel with $h_{1}=1$, $h_{2}=0.6$, $\tau_{1}=0$, $\tau_{2}=1$ and the speed of the train of 30 m/s is considered, and one expected source and one interference source exist with the same parameters as in the previous experiments.

**Figure 6:**
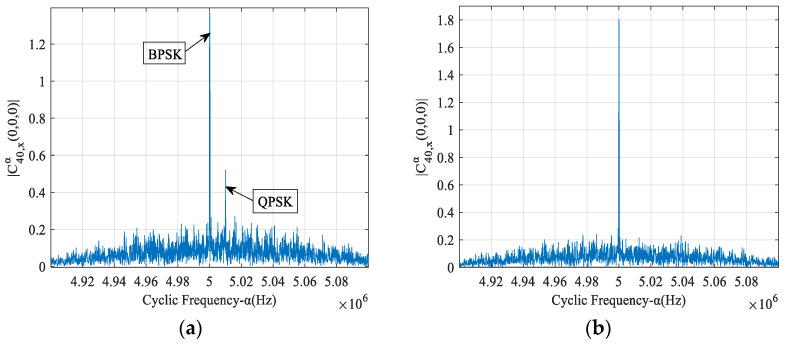
FOCC of received signal with multipath and Doppler effect. (**a**) The case of adjacent-frequency interference. (**b**) The case of same-frequency interference.

The FOCC of the adjacent-frequency interference case with BPSK and QPSK modulation is shown in Figure 6a, where the power of both signals is normalized. Compared with the results of the single-path case in Figure 2a, the amplitude decreases at carrier frequencies and increases at other frequencies due to the multipath effect. The carrier frequencies have changed due to the Doppler effect, but the changes are only 10^−7^ of the carrier frequencies and are not obvious in the figure. The FOCC of the same-frequency interference case with BPSK and QPSK modulation is shown in Figure 6b. Compared with the result in Figure 3a, it can be seen that the FOCC is affected by the multipath propagation and almost not affected by the Doppler.

**Figure 7:**
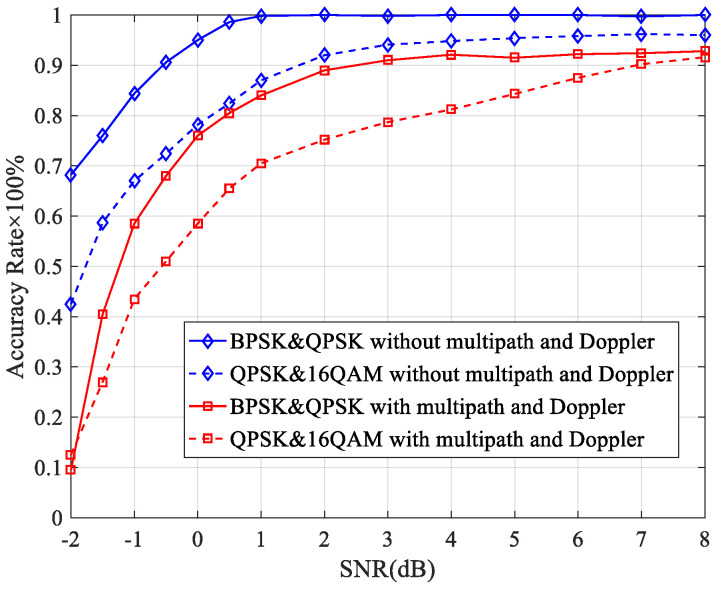
Correct rate of the adjacent-frequency interference detection algorithm in multipath and Doppler environments.

The correct rate of the adjacent-frequency interference detection algorithm in multipath and Doppler environments is shown in Figure 7, with single-path results shown for comparison. It can be seen that the correct rate in a multipath channel is worse than the results in a single path. The reason is that the amplitude of the FOCC at the carrier frequencies decreases due to the power reduction of each signal component when there is a multipath. And the amplitude increases in non-carrier frequencies due to multipath interference, which results in a decrease in the accuracy of the detection of the number of carriers. For low-order modulation, such as BPSK&QPSK, the correct rate decreases from 100% to 91% at SNR = 3 dB. For high-order modulation, such as QPSK&16 QAM, the correct rate decreases from 95% to 89% at the same SNR. These results indicate that the adjacent-interference detection algorithm based on the FOCC is also applicable under multipath and Doppler channels.

**Figure 8:**
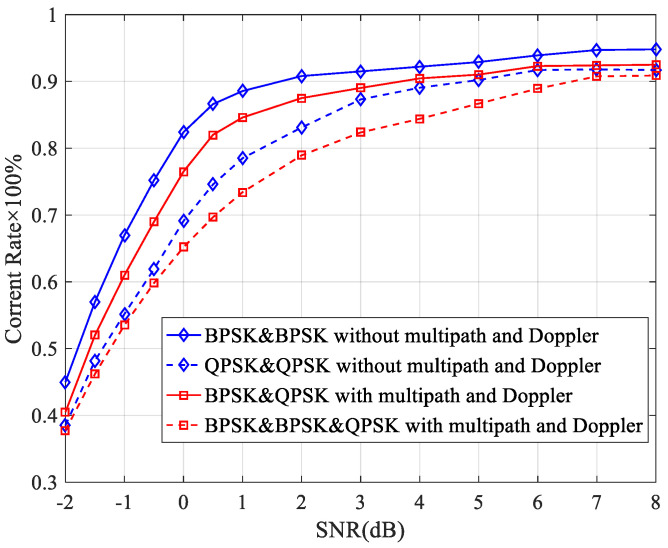
Correct rate of the same-frequency interference detection algorithm in multipath and Doppler environments.

The correct rate of the same-frequency interference detection algorithm in multipath and Doppler environments is shown in Figure 8. It shows that the multipath has a large impact on the same-frequency interference detection algorithm when compared to the results in Figure 5. This is because the signal components propagated by the multipath can be considered as new sources of interference, and the relationship between the FOCC and the received power is changed. Even so, the correct rate is more than 80% when the SNR is greater than 3 dB and can reach 90% when the SNR is greater than 7 dB. This ensures that the algorithm is available in practice.

## 5. Conclusions

For rail transit wireless communication systems, the interference signal has a great impact on the normal operation of trains. Minor interference will lead to a decrease in throughput, and severe interference will lead to communication interruption and train shutdown. In this paper, two detection algorithms are proposed for both adjacent-frequency interference and same-frequency interference, and simulation results validate their effectiveness. To reduce the influence of noise, the FOCC of the received signal is analyzed, and the theoretical derivation for different modulation modes, including BPSK, QPSK and 16 QAM, is performed. The results show that adjacent-frequency interference can be detected by identifying the number of spectral lines that is higher than a preset threshold, and the correct rate is more than 90% when one interference is present and the SNR is greater than 2 dB. Same-frequency interference can be detected based on the relationship between the FOCC and received power, and the correct rate is more than 90% when one interference is present and the SNR is greater than −4 dB. Compared with the traditional second-order statistical methods, the proposed method can detect both adjacent-frequency interference and same-frequency interference. In addition, the performance of both algorithms is affected by multipath and Doppler propagation. However, the correct rate can reach 90% when the SNR is greater than 7 dB. Thus, interference detection based on the FOCC is proved to be effective for the wireless communication of rail transit systems.

## Figures and Tables

**Table 1:** Relationship between the FOCC and the received power of the received signal.

	Without Interference	With One BPSK Interference	With One QPSK Interference
Expected signal is BPSK	$C_{40,x}^{\alpha =f_{ci}}=2k_{1}^{2}=2k^{2}$	$C_{40,x}^{\alpha =f_{ci}}=2(k_{1}^{2}+k_{2}^{2})$	$C_{40,x}^{\alpha =f_{ci}}=2k_{1}^{2}+k_{2}^{2}$
Expected signal is QPSK	$C_{40,x}^{\alpha =f_{ci}}=k_{{}^{1}}^{2}=k^{2}$	$C_{40,x}^{\alpha =f_{ci}}=k_{1}^{2}+2k_{2}^{2}$	$C_{40,x}^{\alpha =f_{ci}}=k_{1}^{2}+k_{2}^{2}$
Expected signal is 16 QAM	$C_{40,x}^{\alpha =f_{ci}}=k_{{}^{1}}^{2}=0.68k^{2}$	$C_{40,x}^{\alpha =f_{ci}}=0.68k_{1}^{2}+2k_{2}^{2}$	$C_{40,x}^{\alpha =f_{ci}}=0.68k_{1}^{2}+k_{2}^{2}$

**Table 2:** Computational complexity comparison of three methods.

Methods	Statistics Calculation	Interference Decision
The proposed algorithm	*O*(*MN*^5^)	*O*(*M*)
Energy detector in Ref. [10]	*O*(*N*^2^)	*O*(*N*^2^)
CME algorithm in Ref. [12]	*O*(*N*log*N*)	*O*(*PN*)

## Data Availability

Not applicable.
